# Normalization of large-scale behavioural data collected from zebrafish

**DOI:** 10.1371/journal.pone.0212234

**Published:** 2019-02-15

**Authors:** Rui Xie, Mengrui Zhang, Prahatha Venkatraman, Xinlian Zhang, Gaonan Zhang, Robert Carmer, Skylar A. Kantola, Chi Pui Pang, Ping Ma, Mingzhi Zhang, Wenxuan Zhong, Yuk Fai Leung

**Affiliations:** 1 Department of Statistics, University of Georgia, Athens, Georgia, United States of America; 2 Department of Biological Sciences, Purdue University, West Lafayette, Indiana, United States of America; 3 Department of Statistics, Purdue University, West Lafayette, Indiana, United States of America; 4 Department of Ophthalmology and Visual Sciences, Chinese University of Hong Kong, Hong Kong SAR, China; 5 Joint Shantou International Eye Center of Shantou University and The Chinese University of Hong Kong, Shantou, China; 6 Department of Biochemistry and Molecular Biology, Indiana University School of Medicine Lafayette, West Lafayette, Indiana, United States of America; 7 Purdue Institute for Integrative Neuroscience, Purdue University, West Lafayette, Indiana, United States of America; 8 Purdue Institute for Drug Discovery, Purdue University, West Lafayette, Indiana, United States of America; University Zürich, SWITZERLAND

## Abstract

Many contemporary neuroscience experiments utilize high-throughput approaches to simultaneously collect behavioural data from many animals. The resulting data are often complex in structure and are subjected to systematic biases, which require new approaches for analysis and normalization. This study addressed the normalization need by establishing an approach based on linear-regression modeling. The model was established using a dataset of visual motor response (VMR) obtained from several strains of wild-type (WT) zebrafish collected at multiple stages of development. The VMR is a locomotor response triggered by drastic light change, and is commonly measured repeatedly from multiple larvae arrayed in 96-well plates. This assay is subjected to several systematic variations. For example, the light emitted by the machine varies slightly from well to well. In addition to the light-intensity variation, biological replication also created batch-batch variation. These systematic variations may result in differences in the VMR and must be normalized. Our normalization approach explicitly modeled the effect of these systematic variations on VMR. It also normalized the activity profiles of different conditions to a common baseline. Our approach is versatile, as it can incorporate different normalization needs as separate factors. The versatility was demonstrated by an integrated normalization of three factors: light-intensity variation, batch-batch variation and baseline. After normalization, new biological insights were revealed from the data. For example, we found larvae of TL strain at 6 days post-fertilization (dpf) responded to light onset much stronger than the 9-dpf larvae, whereas previous analysis without normalization shows that their responses were relatively comparable. By removing systematic variations, our model-based normalization can facilitate downstream statistical comparisons and aid detecting true biological differences in high-throughput studies of neurobehaviour.

## Introduction

Neuroscience research has been revolutionized by experimental approaches that can collect behavioural data simultaneously from multiple individual animals, including worms[1], fruit flies[2], rodents[3] and zebrafish[4]. When these animals are also perturbed by genetical or pharmacological means, their resulting behavioural data would reveal the underlying neural circuitry that drives the behaviour[4, 5], or reveal new drugs for treating neurological diseases[3, 4, 6]. However, these behavioural data are complex in structure and pose many challenges to data analysis. These challenges must be resolved by appropriate statistical approaches to extract accurate information from the behavioural data.

To illustrate the data complexity and analytical challenges, we will outline a popular high-throughput approach for analysing zebrafish behaviour, the visual motor response (VMR). This is a locomotor response displayed by zebrafish larvae upon drastic light onset (Light-On) or offset (Light-Off)[6–9]. In a typical VMR experiment, zebrafish larvae are arranged in a 96-well plate and stimulated by a controlled light source in a lightproof chamber. These larvae can have different genotype or are exposed to different chemical treatments. Their resulting swimming activities are recorded and summarized as number of detected pixels moved in successive frames in the video, or as absolute displacement[10]. These larvae are usually subjected to multiple trials of Light-On and Light-Off over the course of a long period of time (i.e. technical repeats). The experiment is often repeated using independent samples (i.e. biological repeats). The activity of larvae is then extracted from the video, which in turn results in a huge matrix of activity values of many larvae over time.

This experimental design poses challenges to data analysis by traditional statistical approaches including t-test and ANOVA[11] because they cannot not handle time-series data (i.e. data with time-dependency). Consequently, the VMR data have been analysed by advance approaches including repeated-measured ANOVA[12–15] that can handle samples that are repeatedly measured and correlated in time. Our group has also established Hotelling’s T-squared test[10], multivariate analysis of variance (MANOVA)[10], and generalized linear mixed model (GLMM)[16] for VMR data analysis. These approaches take into consideration of unique features of VMR data, such as time dependency among the VMR of individual animals and joint property of all VMR profiles. They also incorporate potential sources of batch effect in the analysis, and allow for proper comparisons between different sample groups.

These analyses, however, do not address another intrinsic issue of VMR data: these data are collected from individual larvae subjected to systematic variations that require normalization. For example, under a particular intensity setting of stimulating light, the larvae in different wells of the 96-well plate may receive slightly different light intensities from the machine. This issue is created by the physical constraint of light generation. Inside the machine, the stimulating light is generated by arrayed LEDs. Since they generate light as point source, they will not evenly illuminate all wells even with a diffuser. When the larvae in the plate are exposed to slightly different light intensities, their resulting VMR may be slightly different. Another example of systematic variation is biological replication. When an experiment is repeated, the biological samples may subject to unwanted variations, including day-day variation in the quality of the embryos, even when they are collected from the same parents. These systematic variations must be corrected by normalization, an approach to adjust values measured on different scales to the same scale for meaningful comparisons between different conditions. In this study, we present a normalization approach for VMR data based on linear-regression modeling. This model-based normalization handles different types of systematic biases separately or together, which allows users to choose specific variations to normalize in their studies. This approach complements the aforementioned statistical analyses for VMR data. Together, they establish an essential framework for analysing high-throughput behavioural data with a similar structure.

## Materials and methods

### Experimental data

The VMR data analysed in this paper were previously collected[10] and were downloaded from the Harvard Dataverse <http://dx.doi.org/10.7910/DVN/HTXXKW>. The dataset comprises activities collected from three wild-type (WT) zebrafish strains: AB, TL and TLAB. For each strain, the VMR data were collected daily from 3 days post-fertilization (dpf) to 9 dpf, using a standard experimental scheme (see S1 Fig)[7, 8, 10, 17–19]. In this scheme, the larvae were arrayed in a 96-well plate. The plate was placed in a Zebrabox system (ViewPoint Life Sciences, Lyon, France) and received light stimulus from a light-controlling unit positioned under the plate. The light intensity of each well was measured by an ILT950 spectrometer (International Light Technologies, Peabody, MA). During an experiment, the plate was first dark-adapted for 3.5 hours (hrs). It was then subjected to three consecutive periods of light onset (Light-On) and light offset (Light-Off). Each of those periods lasted for 30 minutes (mins). Several variables that might affect larval activities were controlled[10]. For instance, all experiments were conducted at the same time of the day with the same type of 96-well plate. Each strain was also individually analysed on separate plates. The research protocol was reviewed and approved by the Purdue Animal Care and Use Committee (PACUC). The approved protocol number is 1201000592.

### Statistical analysis

#### Activity summarization

The larval activity was summarized as Burst Duration, the fraction of frames in each second of the video data that a larva moved[10]. The larvae were first registered by the recoding software in the video frame as grey pixels. These pixels were compared between different frames. A larva was declared moving in a frame if their registered pixels moved more than a preset threshold. The activity for each larva (i.e. Burst Duration) was reported as the fraction of moving frames in each second.

#### Statistical model for data normalization

The normalization in this study was done by linear-regression model. We will first define the group and explanatory variables in the model, and then describe the general framework of the model.

**Group and explanatory variables**: Group variables were used to indicate different normalization conditions in the model, so that normalization can be conducted for each condition separately or for all conditions together. The group variables used in the normalization model include biological variations—Strains: AB, TL and TLAB; and Stage: 3–9 dpf. The group variables also include technical repeats—three consecutive periods of light onset (Light-On) and light offset (Light-Off).

The main explanatory variables are: (1) light intensity: measured from each well of the 96-well plate, and (2) biological replicates: two biological replicates were conducted for each experiment.

**Linear-regression model**: The linear-regression model has the following general form:

$$y_{ij}\;=\;x_{ij}^{T}\beta_{j}+\epsilon_{ij},$$ (1)

where *y*_*ij*_ denotes the observed activity of the *i*th zebrafish larva in group *j* for *i* = 1,…,*n*_*j*_; ***x***_*ij*_ denotes a column vector of explanatory variables for the corresponding larva; ***β****j* represents a column vector containing the parameters of the linear-regression model for the group *j*, and *ϵ*_*ij*_ is the error term. The group *j* is coded to analyze corresponding specific subset of the data. For example, when *j* = strain-AB & Light-On, the model used the observations from the AB strain during the Light-On period for normalization. Our model also assumed a simple linear relationship between the response and predictors. The statistical model was analysed using R software version 3.4.2 <https://www.r-project.org>. The analysis computing scripts can be found at the GitHub repository <https://github.com/zhanzmr/Normalization_Zebrafish>.

#### Evaluation methods for data normalization

We used principal component analysis (PCA) and t-distributed stochastic neighbor embedding (t-SNE) to evaluate the results of the data normalization, as described below.

**Principal Component Analysis** (PCA)[20] is a statistical multivariate analysis tool for dimensionality reduction and data visualisation. PCA takes the possibly correlated multivariate data matrix as input, uses an orthogonal transformation to produce a set of linearly independent output called principal components (PCs). This transformation projects the high-dimensional data in to a low-dimensional space composed of PCs. PCA defines a new orthogonal coordinate system that best describes the intrinsic variability of the data. The variability contains the statistical information of the data set that we need to retain during the normalization procedure. Usually, the high-dimensional data can be visualised by the plotting the first two or three PCs, which usually capture much of the total variability of the data. In the PCA plot, the shape and relative locations of the data points represent the variability of the original multivariate data, and should not substantially change in a good normalization procedure.

In this study, we used PCA to analyse the multivariate VMR data before and after the integrated normalization. The data consist of the time-series activity profiles of individual larva from different stages from 2 seconds before light onset to 3 seconds after light onset. The multivariate VMR data (*X*) were orthogonally transformed by eigendecomposition, which aims to find an orthonormal matrix *P* where *Y* = *PX* such that $S_{Y}\;\equiv \;\frac{1}{n-1}YY^{T}$ is diagonalized. The principal components of *X* are the rows of *P*, or equivalently the eigenvectors of *XX*^*T*^. The PCA results were plotted in a 2D-PCA plot using the first two PCs. Each sample point on the plot represented the activity time profile of one individual larva and was also coloured according to its corresponding developmental stage. The PCA analysis was implemented using R software version 3.4.2 <https://www.r-project.org>.

**t-distributed stochastic neighbor embedding** (t-SNE) is a dimensionality reduction and visualisation tool designed to aid the analysis of multivariate data[21]. It uses stochastic neighbor embedding, a nonlinear transformation, to reduce the dimension of the data. This method visualises the high-dimensional data by giving each sample point a location in a two-dimensional map, which can potentially reveal underlying relationship between data points as clusters.

We used the same data as in the PCA analysis for t-SNE analysis with parameter perplexity equals to 30. The data consist of the time-series activity profiles of individual larva from different stages from 2 seconds before light onset to 3 seconds after light onset. The main algorithm of t-SNE consists of the following steps: First, we constructed the probability distribution of pair-wise similarity between any pair of samples to define the neighbors for each sample. Similar samples had a higher probability to be picked, while dissimilar points had a lower probability to be picked. Second, in the low-dimensional map of t-SNE, we defined a similar probability distribution for the low-dimensional points similar to each other. Finally, we iteratively improved the low-dimensional representation to minimize the Kullback–Leibler divergence between the two distributions so that they looked as closely alike as possible. The results were then plotted on 2D t-SNE map with each point representing one individual larva and coloured according to its corresponding developmental stage. The t-SNE analysis was implemented using R software version 3.4.2 <https://www.r-project.org> with the R package “Rtsne”.

## Results

In this study, we used the linear-regression model to conduct normalization of VMR data. We will first outline the approach for normalization of three different needs, and then illustrate how to integrate several normalizations needs together in an integrated analysis.

### Example 1: Normalization of larval activities obtained from individual wells of a 96-well plate

**Fig 1:**
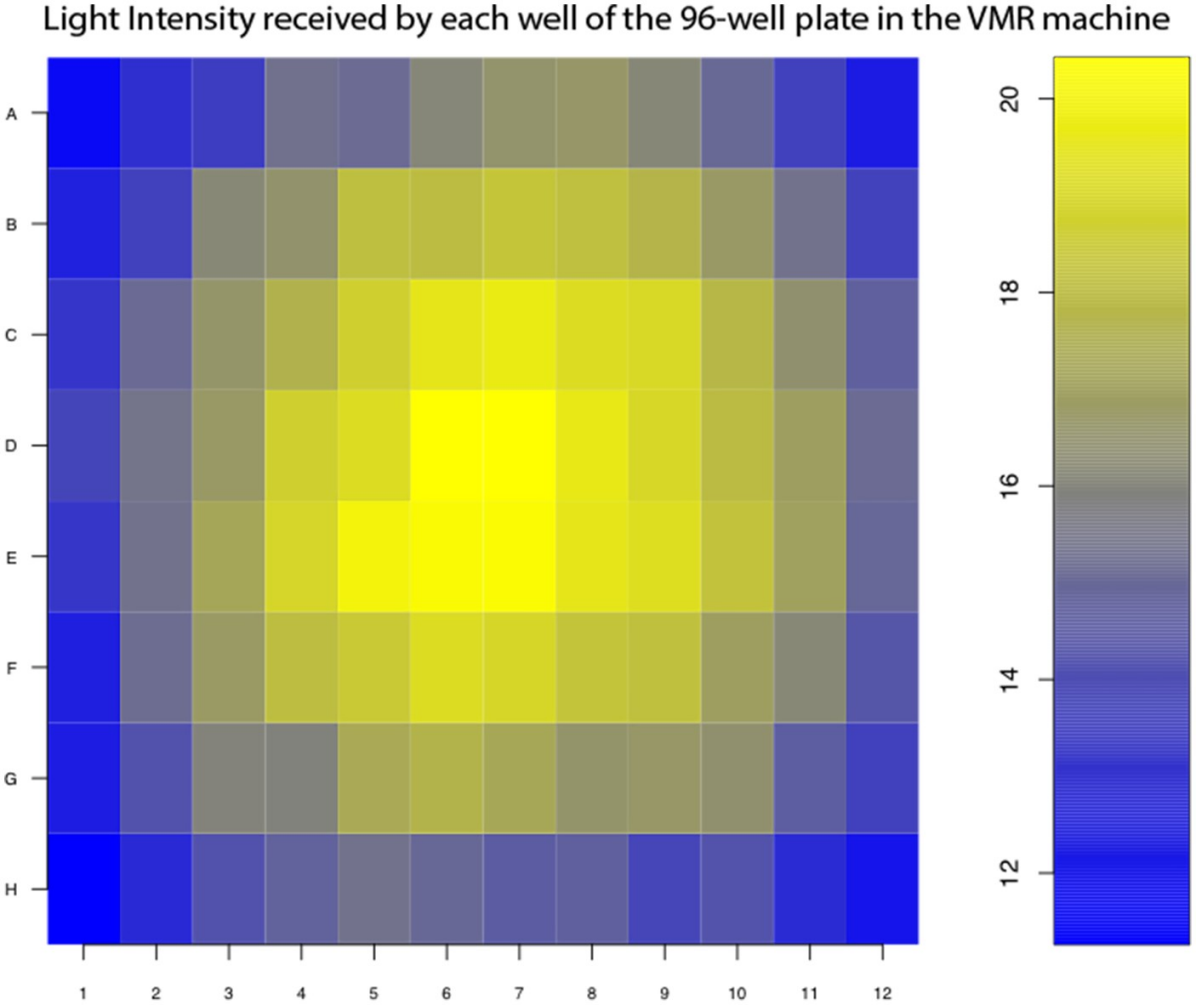
Light-intensity received by each well of the 96-well plate in the VMR machine. The heat map indicates the light intensity (in W/m^2^) received by each of the 96-well pate in the VMR machine, when the light-intensity output was set at 100%. The higher light intensities are represented by yellow colours, whereas the lower light intensities are represented by blue colours.

In the VMR experiment, zebrafish larvae were arrayed individually in different wells of the 96-well plate. They were then subjected light stimulation emitted by the light-controlling unit with LED arrays. Since these LEDs were point light source, the larvae in different wells would receive slightly different light intensities, even though the emitted light was scattered by a diffuser. To illustrate the light variation, we measured the light intensities received in the wells of the 96-well plate when the light-intensity output of the machine was set at 100% (Fig 1). The wells in the center received higher light intensity than those in the corners. This difference in light intensities likely initiated the larvae to display a different level of VMR. Since this difference was not caused by biological difference, it must be removed by proper normalization for downstream analysis. To estimate the effect of light-intensity variation between different wells on VMR, we fit a linear-regression model (1) as follows:

$${activity}_{ij}\;=\;\beta_{0j}+\beta_{1j}light.intensity_{ij}+\epsilon_{ij},$$ (2)

where *activity* is the observed VMR, *i* denotes the *i*th observation (i.e. larva), *j* denotes the group number (i.e. strain, stage, and technical repeats), and *light*.*intensity* is the value of predictor variable for light intensity. The parameters of the model are *β*_0*j*_ and *β*_1*j*_. The parameter *β*_0*j*_ is the intercept of the regression line of the group *j*, which represents the mean response *E*(*activity*)_*j*_ when light intensity is zero. The parameter *β*_1*j*_ is the slope of the regression line, which indicates the light intensity effect for the group *j*, i.e. the change in the mean activity *E*(*activity*)_*j*_ per unit increase in light intensity for group *j*. The random error term for the *i*th observation and *j*th group is denoted as *ϵ*_*ij*_, which is the deviation of the observed activity from the (unobservable) mean activity. This model estimated the effect of light-intensity variation between different wells in the 96-well plate on VMR for all different groups.

Then, we calculated the regression residual, the difference between the observed VMR (*activity*_*ij*_) and the estimated activity caused by light-intensity variation across different wells $({\hat{\text{light-activity}}}_{ij}).$ This residual represents the normalized activity of the *i*th larvae from *j*th group after removing the light-intensity effect from different wells. This subtraction would occasionally introduce negative activity values, which were corrected by adding an offset value *μ*_*offset*_ to keep all normalized activities positive: $\mu_{offset}\geq |\text{min}\;{\hat{\text{(light-activity}}}_{ij})|$ for all *i*,*j*. Together, these calculations would yield ${light-normalized-activity}_{ij}\;(i.e.\;{activity}_{ij}\;-{\hat{\text{light-activity}}}_{ij}+\mu_{offset})$, which could be used for downstream analysis.

**Fig 2:**
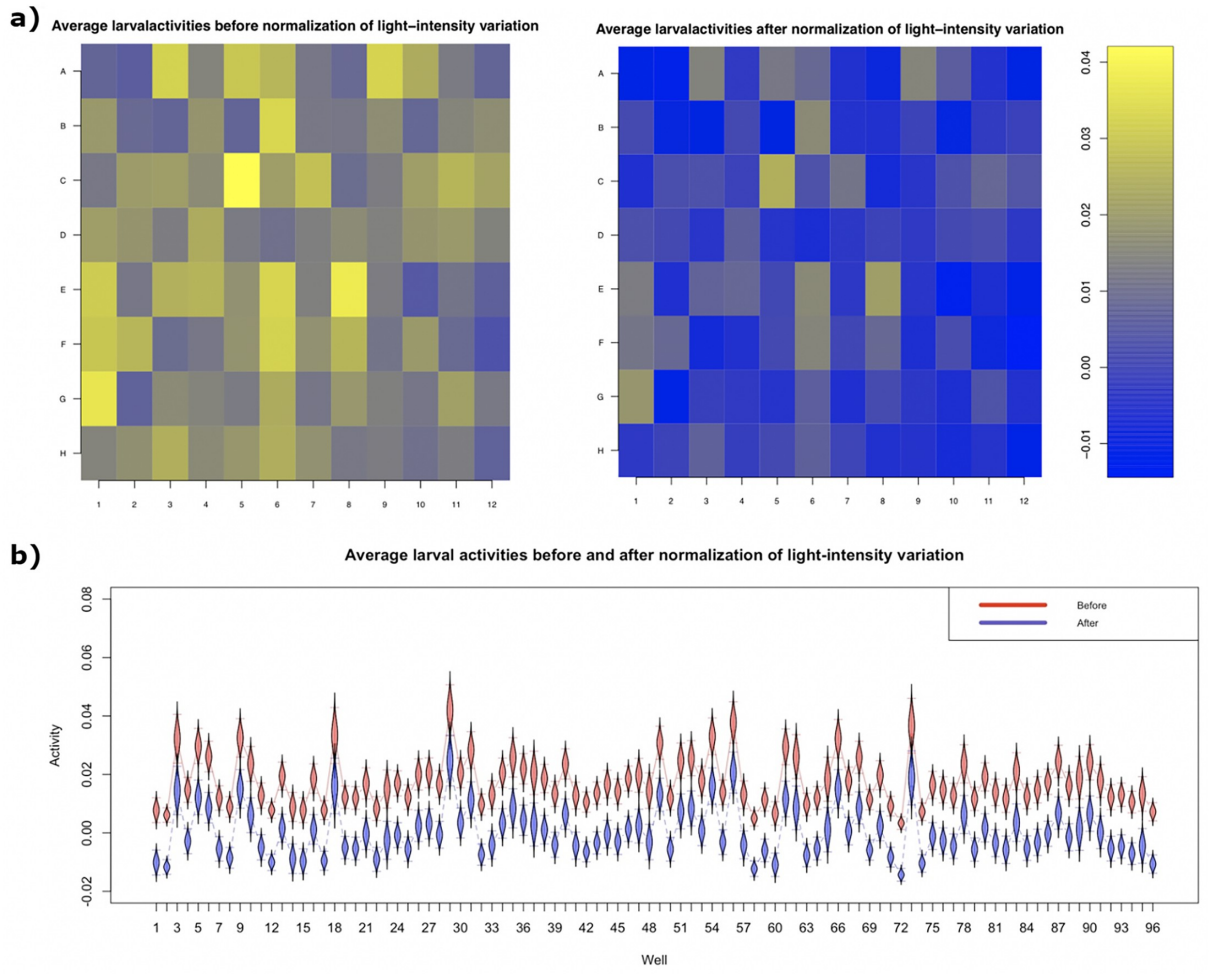
Normalization of larval activities due to variation of light intensity across different wells. (a) Heatmaps showing average larval activities in each well of the 96-well plate before (left) and after (right) normalizing the light-intensity variation across the plate. These larval activities were extracted from 6-dpf TL larvae from 1 to 30s after light onset. (b) A boxplot of average larval activities before (red) and after (blue) light-intensity normalization. The whiskers of each box indicate the 95% confidence intervals.

In this example, we fit the linear-regression model (2) with the VMR data obtained from 6-dpf TL larvae from -30 s to 30 s after light onset (i.e. Light-On Stimulus Trials (yellow bars) in S1 Fig). This group of data was denoted as *j* = 1 in the estimated model:

$${\hat{\text{light-activity}}}_{i1}\;=\;1.907\;\times \;10^{-2}-1.998\;\times 10^{-4}light.intensity_{i1},$$ (3)

where ${\hat{\text{light-activity}}}_{i1}$ is the estimated activity for *i*th observation and group 1, the estimates of the regression coefficients are: $\hat{\beta_{01}}\;=\;1.907\;\times \;10^{-2}$ with standard errors $s(\hat{\beta_{01}})\;=\;3.715\;\times 10^{-4};$ $\hat{\beta_{11}}\;=\;-1.998\;\times 10^{-4}\;$ with standard errors $s(\hat{\beta_{11}})\;=\;3.174\;\times 10^{-5}$. Since the slope coefficient of the light intensity, $\hat{\beta_{11}}$,was significantly different from zero (p-value = 3.12 × 10^−10^), the variation of light intensity across wells positively influenced larval VMR. The fitted model effectively normalized and removed the effect of light-intensity variation on larval activities, which became more uniformly distributed across the 96-well plate (Fig 2).

**Fig 3:**
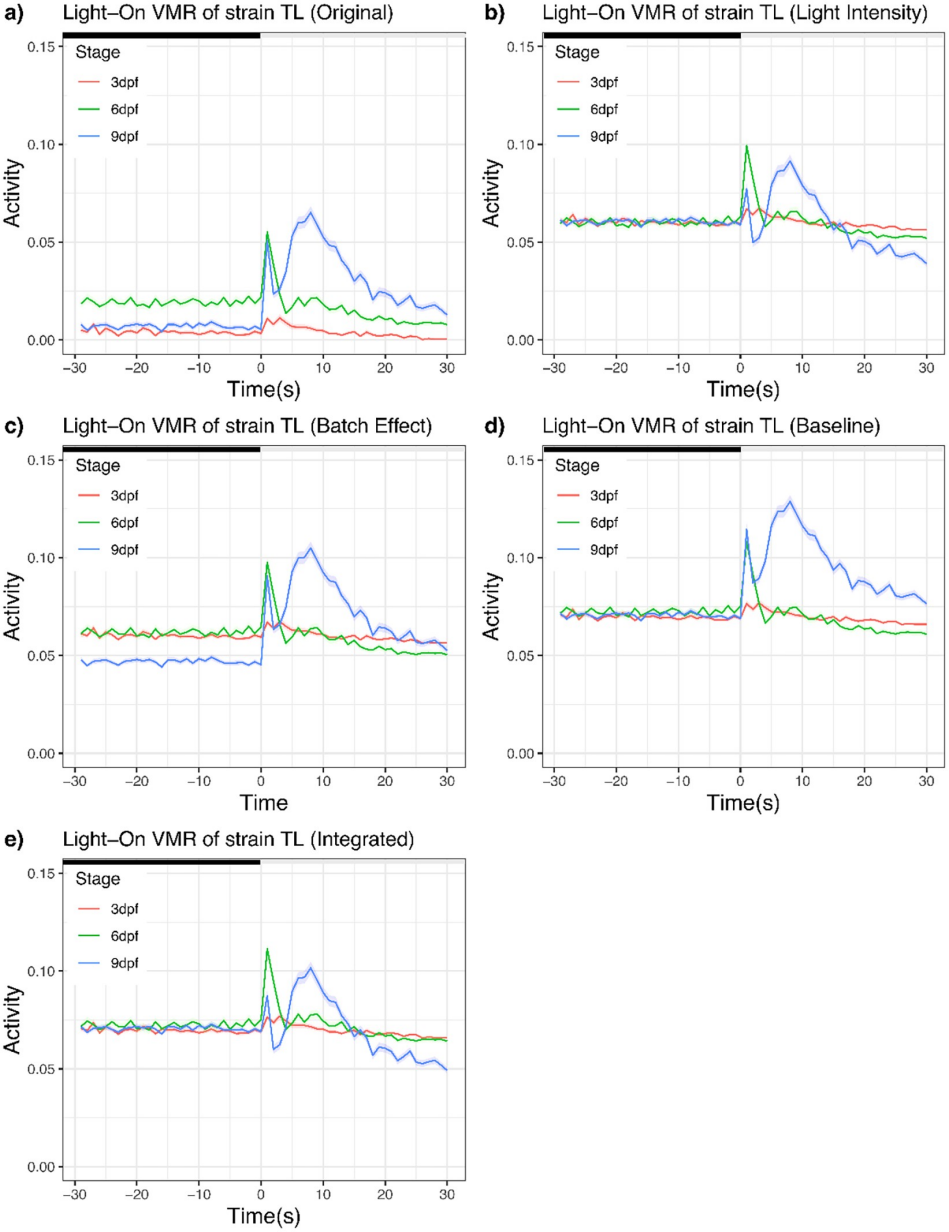
Light-On VMR of strain TL normalized by different approaches. (a) Original data without any normalization. (b) Light-intensity normalization. (c) Batch-effect normalization. (d) Baseline normalization. (e) Integrated normalization. In all plots, the activities of larvae at different stages were plotted from 30 seconds before light onset to 30 seconds after light onset. The solid traces show the mean activities (red trace: 3dpf; green trace: 6 dpf; and blue trace: 9 dpf), whereas the ribbons surrounding these activity traces indicate the corresponding standard error of the pointwise mean activity. The offset value *μ*_offset_ for (b) to (e) was 0.06.

To illustrate how this light-intensity normalization affects the activity profile, we plotted a subset of data before and after normalization (Fig 3a & 3b). In this dataset, we used Light-On VMR data of TL strain from three different stages:3 dpf, 6 dpf and 9 dpf. Their light-intensity normalization was done separately. The activities from all replicates were averaged and plotted. Before normalization, both 6-dpf larvae and 9-dpf larvae displayed similar peak activities right after light onset, whereas 3-dpf larvae displayed little activity. After normalization, the 6-dpf larvae displayed a noticeably higher peak activity after light onset than the 9-dpf larvae, whose peak activity was relatively comparable to that from the 3-dpf larvae at the same period.

### Example 2: Normalization of batch effect

Many VMR experiments require the analysis of more than 96 larvae, exceeding the capacity of a 96-well plate that would be analysed in the same VMR machine per run. Consequently, these larvae were analysed sequentially on different 96-well plates in the same VMR machine, or in parallel in different VMR machines. These experimental schemes created batch variations on larval activity, which can be normalized by the following linear-regression model:

$${activity}_{ij}\;=\;\beta_{0j}+\beta_{kj}I(batch\;k)+\epsilon_{ij},\;k\;=\;1,\ldots ,K$$ (4)

where *activity* is the response, *i* is the *i*th observation (i.e. larva), *j* is the group number (i.e. strain, stage, and technical repeats), and *I*(*batch k*) is the indicator function such that *I*(*batch k*) = 1 when the activity data come from batch *k*, otherwise *I*(*batch k*) = 0. We assume there are *K* levels of batch effect to be removed. The parameters of the model are *β*_0*j*_ and *β*_*kj*_ for *k* = 1,…,*K*, and *ϵ*_*ij*_ denotes the random error. The parameter *β*_0*j*_ is the grand mean, which represents the mean normalized activity, *E*(*activity*)_*j*_, for all observations from group *j*; the parameter *β*_*kj*_ is the batch effect for batch *k*, which is the deviation from the grand mean due to the batch effect of batch *k* for the group *j*.

To illustrate our approach for normalizing batch effect, we analysed our VMR dataset that contained two biological replicates conducted on different days in the same VMR machine. We used a subset of the VMR data obtained from 6-dpf TL larvae from -30 s to 30 s after light change, where we denoted as group *j* = 1. We modeled the two replicates as two batches: *replicate*1 and *replicate*2. They were treated as separate explanatory variables that took two possible values: 1 and 0. “1” indicated the activity data belong to this replicate, whereas “0” indicated the activity data did not belong to this replicate. Therefore, *replicate*1 + *repliacte*2 = 1 for each pair of *i* and *j*. This subset of data was used to fit a model:

$${\hat{\text{batch-activity}}}_{i1}\;=\;1.207\times 10^{-2}-1.586\times 10^{-3}replicate1_{i1}+1.586\times 10^{-3}replicate2_{i1},$$ (5)

where the ${\hat{\text{batch-activity}}}_{i1}$ is the estimated activity for *i*th observation in group 1. The estimates of the regression coefficients are: $\hat{\beta_{01}}\;=\;1.207\times 10^{-2}$ with standard errors $s(\hat{\beta_{01}})\;=\;2.885\times 10^{-4};$ $\hat{\beta_{11}}\;=\;-1.586s\times 10^{-3}$ with standard errors $s(\hat{\beta_{11}})\;=\;4.090\times 10^{-4}$. Since we implemented the zero-sum constraint on *β*_11_ and *β*_21_, i.e. *β*_11_ + *β*_21_ = 0, we have $\hat{\beta_{21}}\;=\;1.586\times 10^{-3}$. The slope coefficient of the light intensity $\hat{\beta_{11}}$ was significantly different from zero (p-value = 1.05 ×10^−4^), which indicates that the activities from two replicates are significantly different.

Then, we calculated a regression residual to remove the batch effect from biological replicates. It is the difference between the observed VMR (*activity*_*ij*_) and the estimated activity caused by batch-batch variation $({\hat{\text{batch-activity}}}_{ij}$). This residual represents the normalized activity of the *i*th larvae from *j*th group after removing the batch-batch effect. This subtraction would occasionally introduce negative activity values, which were again corrected by adding an offset value *μ*_*offset*_ to make all normalized activities positive: $\mu_{offset}\geq |\text{min}(\;{\hat{\text{batch-activity}}}_{ij})|$ for all *i*,*j*. These calculations would yield ${batch-normalized-activity}_{ij}(i.e.\;{activity}_{ij}\;-{\hat{\text{batch-activity}}}_{ij}+\mu_{offset})$, which could be used for downstream analysis.

To illustrate the effect of batch normalization on the VMR profiles, we again plotted the same Light-On dataset for TL strain (Fig 3c). Compared with the unnormalized data (Fig 3a), the batch-normalized activities now share the same mean activity across time. In other words, if we summarize each curve in Fig 3 into its corresponding mean value, they will have the same mean value after batch normalization.

### Example 3: Normalization to a common baseline

**Fig 4:**
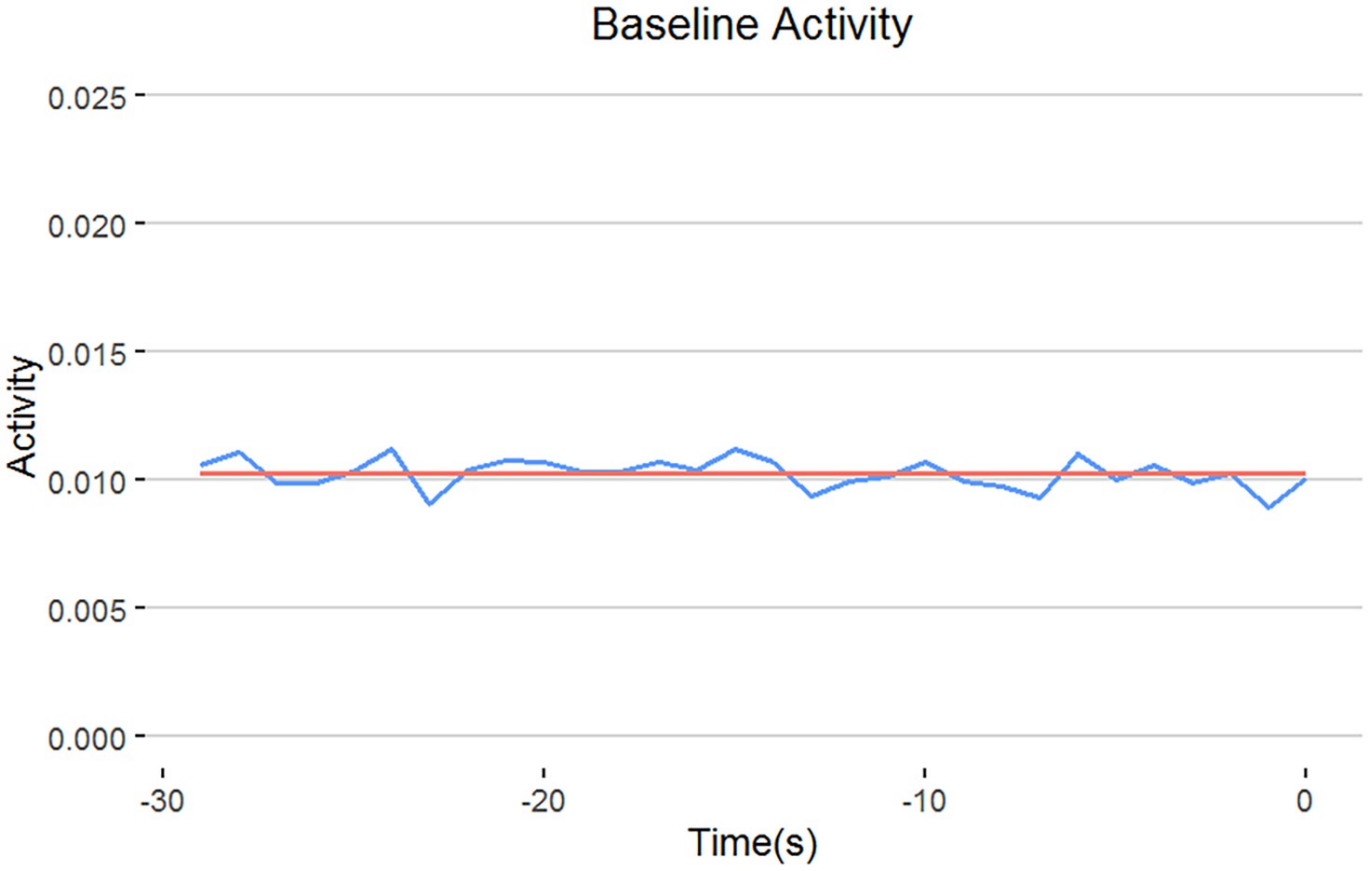
Selection of baseline activity. In this study, we proposed to use the average activity of last 30 seconds from the 3.5-hour adaptation period (i.e. regions under red bar in S1 Fig) as baseline for normalization. The blue line indicates the mean activity for each second, whereas the red line indicates the grand mean of all activities in the whole 30-second period. Since the two lines are highly comparable, this suggests the grand mean of the activities is very stable and can be used for baseline normalization.

We previously designed a Hotelling’s T-squared test[10] to compare VMR between two samples in a specific time frame. One of the most important comparisons was the time frame after light change, as this could reveal the difference in light sensation between groups. This statistical comparison allowed us to evaluate not only visual impairment in fish mutants, but also drug improvement of their impaired vision[6, 17]. However, the success of this comparison relied on an implicit assumption: the two samples displayed comparable activities before light change. In reality, different samples often displayed varying baseline activities (Fig 3a). This baseline variation must be normalized for an effective comparison of two samples by the Hotelling’s T-squared test. The baseline can be the grand mean activity across all conditions from a specific time period immediately before light change, for example the last 30 seconds from the 3.5-hour adaptation period before the light change (i.e. regions under the first red bar in S1 Fig), because the larvae should be acclimatized and would be more stable after several hours of adaptation. In fact, the grand-mean activity 30 seconds before the light change was 0.01024 across all strains and stages (Fig 4, red line). It was around the average activities per individual second during the same 30-second period (Fig 4, blue line), which were stable. Hence, the grand-mean activity could be used for baseline normalization.

The VMR of different groups were then normalized by adjusting the averaged activities of each group to 0.01024. This was achieved by the following steps: First, we obtain a baseline normalization factor by fitting a linear-regression model with only intercept term:

$${activity}_{ij}\;=\;\beta_{baseline\_norm\_factor\;j}+\epsilon_{ij},$$ (6)

where *activity*_*ij*_ is the *i*th observation in the *j*th group. The parameter of the model, *β*_baseline_norm_factor j_, can be estimated as ${\hat{\beta }}_{baseline\_norm\_factor\;j}\;=\;ave(dark_{activity_{j}})-0.01024$, where *ave*(*dark*_*activity*_*j*_) denotes the average activity from the 30-second time period before the light change for group *j*. Then, we calculated a regression residual, the difference between the observed VMR (*activity*_*ij*_) and the baseline normalization factor $({\hat{\beta }}_{baseline\_norm\_factor\;j}$). Since the calculation might yield negative values for activities, we again corrected that by adding an offset value *μ*_*offset*_ to make all baseline normalized activities positive: $\mu_{offset}\geq |\text{min}(\;{\hat{\beta }}_{baseline\_norm\_factor\;j})|$ for all *j*. Together, these calculations would yield ${baseline-normalized-activity}_{ij}\;(i.e.\;{activity}_{ij}-{\hat{\beta }}_{baseline\_norm\_factor,j}+\mu_{offset}$). After this baseline normalization, all groups will have the same group average, 0.01024 + *μ*_*offset*_, as the baseline (Fig 3d). These baseline-normalized activities could be used to perform the Hotelling’s T-squared test.

### Integrated normalization of VMR data

In the previous sections, we demonstrated how to normalize different variables of the VMR experiments by linear-regression models. In practice, these variables should be normalized all at once. The resulting residuals from the model would be free from systematic variations and can be used to reveal true biological difference between different samples. To illustrate the value of our normalization approach, we will normalize all three variables outlined in the earlier examples using the same Light-On VMR data of TL strain at 3, 6, and 9 dpf again. The integrated normalization had three steps: First, it normalized light-intensity variations in the 96-well plate; Second, it used the residuals from step 1 as the response variable to normalize the batch effect; Third, it used the residuals from step 2 to perform a baseline normalization. In this step, an offset value *μ*_*offset*_ = 0.06 was applied. The result of integrated normalization is shown in Fig 3e. The normalized data were then used for statistical comparisons by the Hotelling’s T-squared test[10] (Table 1). In this example, we analysed three seconds around the light change to highlight the effect of integrated normalization.

**Table 1:** The results of Hotelling’s T-squared test of Light-On VMR between TL strain at different stages.

Before integrated normalization		
**Comparison**	**Test statistic (p-value)**	
**Stage (dpf)**	**Before light onset (-2–0 s)**	**After light onset (1–3 s)**
**3 vs. 6**	115 (0.0000)	208 (0.000e+00)
**3 vs. 9**	5.83 (0.121)	535.7 (0.000e+00)
**6 vs. 9**	82.8 (0.0000)	13.6 (3.671e-3)
**After integrated normalization**		
**Comparison**	**Test statistic (p-value)**	
**Stage (dpf)**	**Before light onset (-2–0 s)**	**After light onset (1–3 s)**
**3 vs. 6**	13.2 (0.0132)	117.66 (0.000e+00)
**3 vs. 9**	0.188 (0.9795)	66.04 (8.071e-14)
**6 vs. 9**	11.4 (0.0152)	94.02 (0.000e+00)

The top table contains the test results before integrated normalization, whereas the bottom table contains the test results after integrated normalization. The corresponding activity plots can be found in Fig 3a and 3e respectively. In both tables, we presented the comparisons of VMR three seconds before light onset and three seconds after light onset.

Before integrated normalization (Fig 3a), the activity of 6-dpf larvae before light onset was significantly different from that of the 3-dpf and 9-dpf larvae (Table 1, p < 0.0001), whereas the activities of 3-dpf and 9 dpf larvae were comparable (Table 1, p = 0.121). After light onset, the 3-dpf larvae did not display much activities and was significantly different from the 6-dpf larvae and 9-dpf larvae (Table 1, p < 0.0001). The 6-dpf and 9-dpf larvae, however, displayed a strong Light-On VMR in the first three seconds after light onset that were relatively comparable to each other (Fig 3a; Table 1, p < 3.671e-3). The situation was quite different after the integrated normalization (Fig 3e). The normalization brought the activities before light onset to a more comparable level and changed the shape of the activity profiles after light onset. In particular, the peak activity of 6-dpf larvae was now substantially higher than that of the 9-dpf larvae (Table 1, p < 0.0001).

### Evaluation of model-based normalization for VMR data

**Fig 5:**
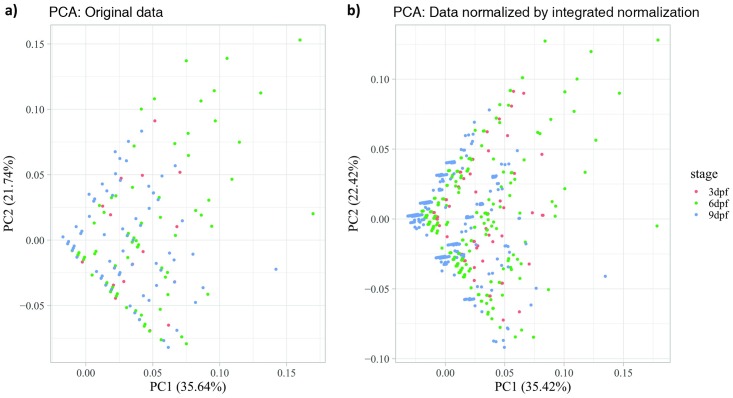
Two-dimensional Principal Component Analysis (2D PCA) scores plots of VMR data before and after the integrated normalization. (a) Original data without normalization. (b) Data normalized by integrated normalization. Both plots include the activities of larvae from 2 seconds before light onset to 3 seconds after light onset at different stages: 3 dpf (red), 6 dpf (green), and 9 dpf (blue). Each dot represents the time-series activity profile of one individual larva from different stages.

**Fig 6:**
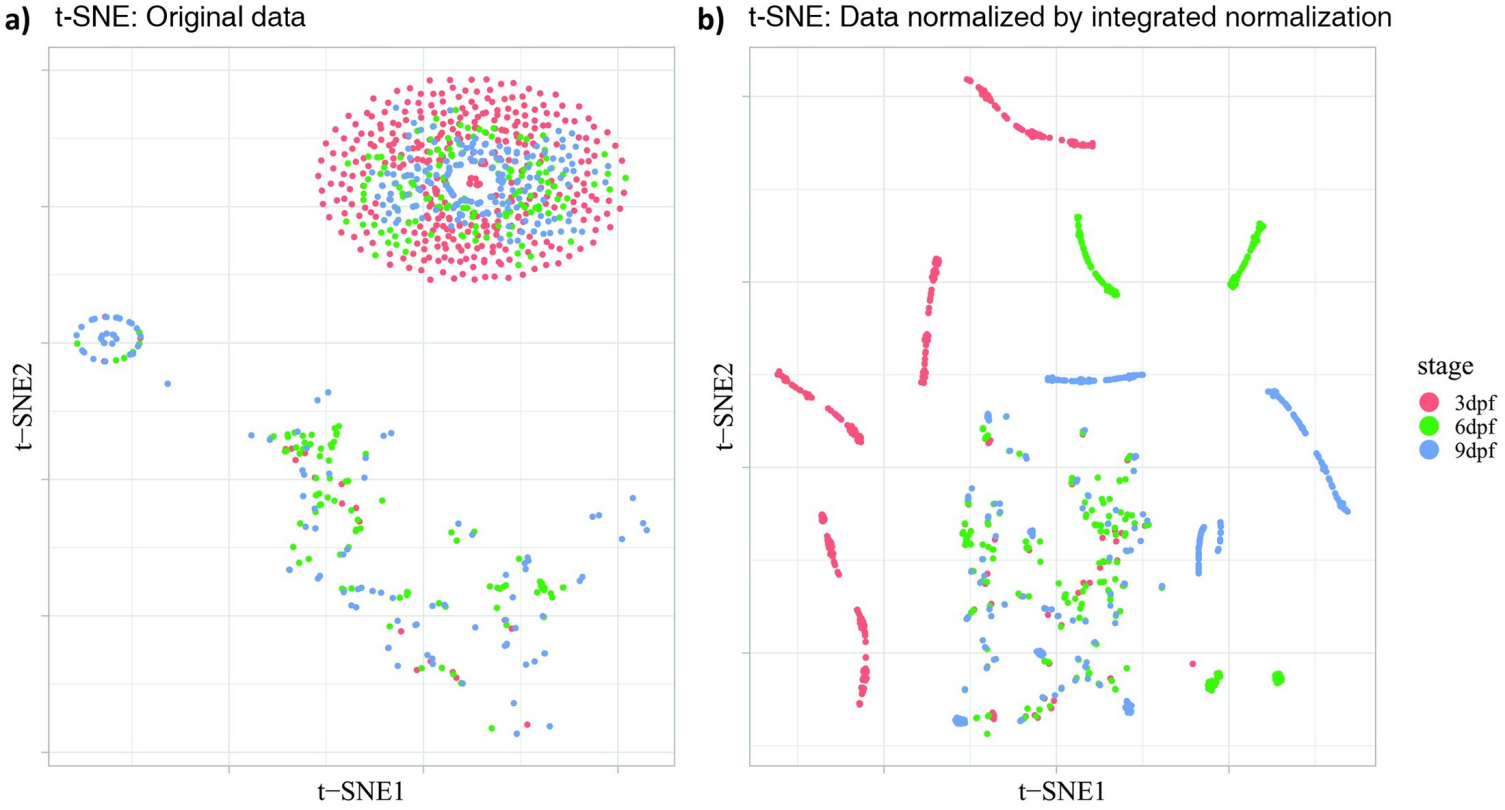
Two-dimensional t-SNE plots of VMR data before and after the integrated normalization. (a) Original data without normalization. (b) Data normalized by integrated normalization. Both plots include the activities of larvae from 2 seconds before light onset to 3 seconds after light onset at different stages: 3 dpf (red), 6 dpf (green), and 9 dpf (blue). Each dot represents the time-series activity profile of one individual larva from different stages.

Any effective normalization approach should demonstrate two properties that would make the normalized data reveal the underlying information better than the original data. First, the normalization approach should not change the intrinsic variability of the data. Data variability is the extent to which sample points vary in a data distribution. The change of data variability is an indicator of whether the normalized data have been distorted or not. Our normalization procedure should maintain data variability since linear-regression modelling focus on the mean of the data. Second, the normalization approach should help find a clear and concrete grouping pattern for data from different classifications. These two properties were integral components of our model-based normalization for VMR data, as illustrated by PCA (Fig 5) and t-SNE (Fig 6).

We visualised the VMR data before and after normalization by PCA. This method transforms the multidimensional data into fewer orthogonal dimensions called principal components (PCs) that are uncorrelated with each other. Each PC captures the largest possible variance compared to the next one. In Fig 5, we plotted the first two PCs that captured more than 55% of the data variance. The plots show that i) the normalized dataset has a similar triangular shape compared to the raw data; ii) the relative location of the individual data points are similar; and iii) the variance explained by PC1 and PC2 are similar before and after normalization (Fig 5a: 35.64% and 21.74% vs. Fig 5b: 35.42% and 22.42%). These together suggest that the intrinsic variability of the data was maintained by our normalization method.

To reveal the clustering of larva from different stages, we further visualised the VMR dataset before and after normalization by t-SNE. This method transforms the multidimensional dataset into low dimensional space by converting the distances in multidimensional space between sample points into probabilities that represent their similarities. In Fig 6, we plotted the 2D t-SNE map. Before normalization, the data points from different developmental stages were either scattered randomly on the plot or were aggregated together (Fig 6a). After normalization, data points from different developmental stages were clearly clustered together and separated from other stages (Fig 6b). There were some data points from different stages aggregated in the middle bottom of the figure, probably reflecting the larvae involved displayed similar behavioural pattern, for example, moving little or not at all during the experimental period. The t-SNE map in Fig 6b shows a clearer clustering of larvae from the same stages that display similar behavioural patterns. This clear clustering of data from similar stages indicate that our normalization approach can reveal patterns in the data closer to the biological nature.

## Discussion

High-throughput approaches for collecting behavioural data have revolutionized neuroscience research, when the collected data are properly analysed. These data are often multi-dimensional, as they are continually and repeatedly collected from multiple individuals under different kinds of perturbations. One such experimental approach is called VMR. This assay collects swimming responses from many zebrafish larvae arranged in 96-well plates over time, which make the data correlated in time and by location. If the data are collected in very short timeframe in seconds, some larvae may not move. The resulting data will then contain many zero values, which creates a data-imbalance problem. These features of the VMR data cannot be dealt with by traditional analyses including the t-test and AVONA. In previous studies, we addressed the time-dependency issue by the Hotelling’s T-squared test[10], and the data-imbalance problem and location-correlation issue by the GLMM[16]. These new analyses enable proper statistical analysis of VMR data for the first time. Nonetheless, these statistical analyses did not address another fundamental issue of these high-throughput behavioural data: the experiments are often subjected to systematic variations. If these variations are not accounted for, they would affect the performance of the aforementioned statistical analyses. To address this analytical gap, we established an approach to normalize the systematic errors by linear-regression modeling.

Our normalization approach modeled the relationship between larval activities (response) and uncontrolled systematic variations (explanatory predictors). The resulting regression residuals were then used as the normalized activities. This approach was flexible because it could easily handle different types of uncontrolled experimental conditions by adding separate terms in the normalization model. For example, it handled continuous variables such as light intensity (Figs 2 and 3b) and baseline activities (Fig 3d), and categorical variables such as biological replicates (Fig 3c) These variables can also be normalized in one integrated model to remove the effect of multiple systematic errors at once (Fig 3e). The linear-regression model can also be adapted to different sample groups (genotypes strains, and/or stages), and enabled normalization of selected subset of data.

By removing systematic biases, new patterns can be revealed from the normalized data. For example, the integrated normalization (Fig 3e) removed the difference in activities due to variation in light intensity between different wells of the 96-well plate. This has changed the activity profile of the individual stages. In addition, the normalization also brought the activities before light onset to a comparable level, essentially assuming that was the baseline activities for different groups of larvae. This assumption may not be applicable to all cases, but it can be used to assess the extent of relative level of larval response upon light onset. In our case, this normalization clearly shows that the 6-dpf TL larvae responded to light simulation much stronger than the 9-dpf TL larvae, a conclusion that can only be drawn after appropriate normalization. The new patterns revealed from the normalized data likely reflect the underlying biological pattern clearer, as the model-based normalization did not alter the data structure (Fig 5), and could cluster data in the same categories better (Fig 6).

Our model-based normalization had several limitations. First, it only handled continuous responses of larval movement. This limitation can be resolved by using generalized linear model to deal with categorical response variable. Second, the linear-regression model mainly focuses on the linear relationship between the response and the explanatory variables. This can be partly resolved by adding higher-order terms of explanatory variables. The model can also be generalized through nonparametric regression techniques, in which no assumption is made on the relationship between response mean and predictors to any specific class, including linear or quadratic class[22]. This approach can be useful to model the effect of light intensity as a function of visual sensitivity which operates over several log units. Third, it does not consider the temporal dependency during the normalization. This can be resolved by adding time-series terms to the linear-regression model or generalizing it to time-series regression model[23].

To conclude, our study has implemented the linear-regression model to normalize VMR data. The normalized data can then be used in downstream analyses including the Hotelling’s T-squared test[10] and the GLMM[16] for statistical comparisons between sample groups. This model-based normalization can be integrated into our framework for VMR data analysis in the following workflow: (1) Normalization using linear-regression model; (2) Comparing the larval activities of different groups using Hotelling’s T-squared test; (3) Using GLMM to model the relationship between responses and candidate predictors; and (4) Combining the results from (2 & 3) to interpret larval activities. This framework facilitates the dissection of the underlying circuitry that drives VMR[24], and in turn the identification of true biological factors that affect the behaviour. We also expect our normalization and analysis framework applies to other high-throughput behavioural data with a similar structure, and can unveil new insights into neurobehaviour.

## Supporting information

S1 Fig VMR experimental scheme. This scheme was used to collect the dataset used in this analysis. In the scheme, the larvae were first dark adapted for 3.5 hrs (long black bar on the left). Then, they were subjected to three consecutive trials of light onset (grey bars) and light offset (short black bars). Each light-on or light-off session lasted for 30 mins. Three technical repeats were also performed in each biological replicate; two biological replicates were performed for each condition. In this study, we extracted the data from 30 s before light change (red bars; not to scale) to 30 s after light change (blue bars; not to scale) for statistical analyses. In some cases, we further restricted the analysis to from 3 s before light change to 3 s after light change. This scheme is modified from Liu et al., 2015[10].
(TIF)
