# Supplementary figures and images for: Normalization of large-scale behavioural data collected from zebrafish
Source: PLoS One. 2019 Feb 15;14(2):e0212234. doi: 10.1371/journal.pone.0212234 (PMC6377122; doi:10.1371/journal.pone.0212234)

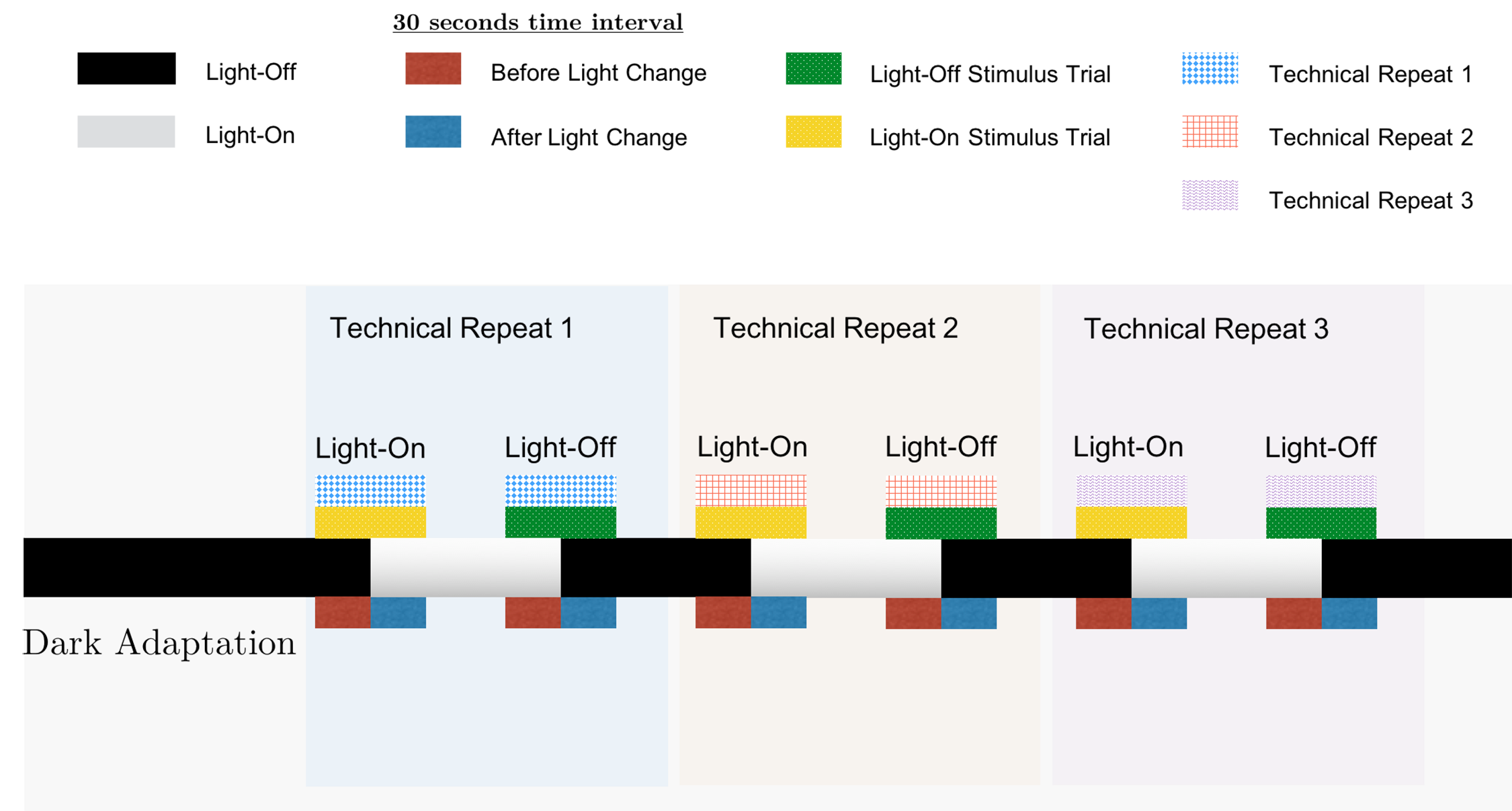

Supplement: S1 Fig — This scheme was used to collect the dataset used in this analysis. In the scheme, the larvae were first dark adapted for 3.5 hrs (long black bar on the left). Then, they were subjected to three consecutive trials of light onset (grey bars) and light offset (short black bars). Each light-on or light-off session lasted for 30 mins. Three technical repeats were also performed in each biological replicate; two biological replicates were performed for each condition. In this study, we extracted the data from 30 s before light change (red bars; not to scale) to 30 s after light change (blue bars; not to scale) for statistical analyses. In some cases, we further restricted the analysis to from 3 s before light change to 3 s after light change. This scheme is modified from Liu et al., 2015[10]. (TIF) [file pone.0212234.s001.tif]
